# NC 4.0, a Novel Approach to Nonconformities Management: Prioritizing Events With Risk Management Tools

**DOI:** 10.3389/frai.2021.752520

**Published:** 2021-11-16

**Authors:** Dvir Ravoy, Yisrael Parmet

**Affiliations:** Industrial Engineering and Management Department, Ben-Gurion University of the Negev, Beersheba, Israel

**Keywords:** nonconformities, risk management, Quality 4.0, events prioritizing, severity levels

## Abstract

Quality 4.0, the correspondent quality practice fit to address the Industry 4.0 mindset, is expected to provide models and processes endorsed by continuous improvement and data-driven proofs, especially given the exponential growth in available data. The research consolidates the reality of big data availability (part of Quality 4.0) with a generic aspect of quality—managing nonconformities. Its purpose is to suggest a model to improve the initiation step for dealing with nonconformity by prioritizing these events. The new concept in the model suggested is incorporating the risk management method of prioritizing into the nonconformity’s management. These tools are designed to transform qualitative data into quantitative ones and enable easier decision-making, in this case, choosing which issue to deal with first. The research approach is developing and testing the suggested model as a pilot in a real production environment to establish its impact and define key guidelines for utilizing it in various processes and, in addition, to conduct a survey among quality experts from different organizations for reference. Two main outcomes were achieved during the research: The quality experts’ survey welcomed the model concept as a structured tool based on the solid risk management methodology. Implementing the model on actual production lines resulted in a significant reduction of NC financial impact as the events were solved as per their impact.

## Introduction

The forth industrial revolution, or Industry 4.0, is the implementation of smart technologies onto traditional manufacturing, changing its capabilities, efficiency, and profit potential altogether (Durana et al., 2019). This change emerged as technologies such as early digital computing and process-driven automation were exhausted to the fullest and new technologies such as big-data, machine learning, and extensive use of analytics became easier to use.

Quality 4.0, the correspondent quality practice fit to address the Industry 4.0 mindset, is expected to provide models and processes endorsed by continuous improvement and data-driven proofs (Zonnenshain and Kenett 2020). In practice, Quality 4.0 initiatives can determine how quickly we inquire, aggregate, and learn from new data; how fast we respond to changes in processes; and how fast we infer about our products’ faults and find root causes (Radziwill 2018). Unfortunately, even though it has the potential to contribute to the success of Industry 4.0 (Stefanović et al., 2019), Quality 4.0 is not standing up for the challenge. The notion of Quality 4.0 is being acknowledged extensively in terms of implications and systems (Stefanović et al., 2019), but the practicality of quality processes is hardly discussed. Furthermore, according to Zonnenshain and Kenett, quality processes have not gained innovative changes for over a decade and a half (Zonnenshain and Kenett 2020). From this we can conclude that quality processes are in need of refitting, to say the least, to the 4.0 age.

One of the core processes in operations is managing nonconformities (NCs), also known as defect management. This is a generic part of any process well defined by the ISO standards (BSI 2009). The NC management process was chosen for research because it holds great interest for the business community in terms of impact on its operation while having academic potential and a need for more detailed models (Ravoy 2018). The NC management process is also notorious for being a heavy data producer (Bacoup et al., 2018), which fits our agenda of data-driven quality engineering.

In this article, we have reviewed one of the key elements at the base of any process effectiveness that is prioritizing (Rezaei et al., 2018). We have proposed a practical concept for prioritizing NCs in order to improve the overall operation effectiveness. The application of this concept is based on the fact that as a part of the Quality 4.0 revolution, the organization collects high volume of data that are not utilized in this aspect. We have developed a model in which we prioritize the NCs that use the same database collected by the organization in each case, thus not burdening the organization and streamlining its activities.

The article is structured as follows—section 2 contains the literature review regarding quality processes, nonconformities, and prioritizing methods; section 3 describes the methods used for the research; and section 4 presents the research results, conclusions and discussion.

## Literature Review

### Processes and Quality Processes Definition

As stated in the introduction, processes and process management are a cornerstone for quality. The Oxford dictionary defines a process as “a series of actions or steps taken in order to achieve a particular end” (Oxford University Press 2016). A wider view of the business or operational process defines the process as a set of interdependent and linked procedures which, at every stage, consume one or more resources (employee time, energy, machines, and money) to convert inputs (data, material, parts, etc.) into outputs. These outputs then serve as inputs for the next stage until a known goal or result is reached. A process operates within a range of set requirements. This range can be developed naturally or be defined by a set of measures and goals, creating a standard (Becker et al., 2014). Basically, a process consists of three main successive phases or steps, input, processing, and output.

The quality of a process is defined in several aspects; two of them are the “output” and “process” stability. In each aspect, there are several questions to answer. In the input aspect, the questions are as follows: How close is the product to the customer requirements? What is the level of profitability of the product? And what is the level of the customer experience from the product? In the process stability aspect, the questions are as follows: How centered is the process compared to its defined range? And how repeatable/predictable is the process? Obviously, an important measure is the process cost, and a better process (stable and less variable) will cost less.

**FIGURE 1:**
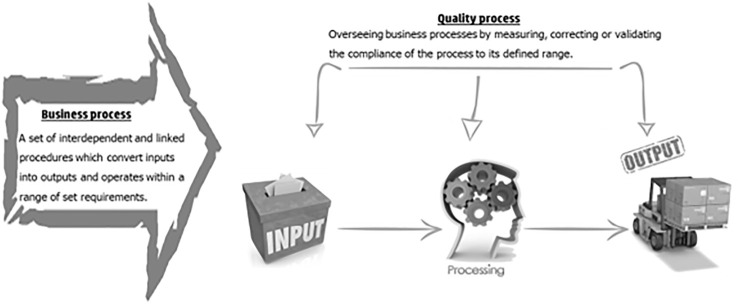
Business process vs. quality process.

The quality process basically has the same three phases as any other process (input–processing–output), but it refers in an orthogonal manner to an operational/business process measuring its stability, level of centralism, etc. In other words, a quality process can be defined as a method or indicator overseeing an operational/business process by measuring, correcting, or validating the compliance of the process to its defined range. The quality processes could be illustrated as perpendicular to the business process as seen in Figure 1.

### Quality Processes Management Approach

**FIGURE 2:**
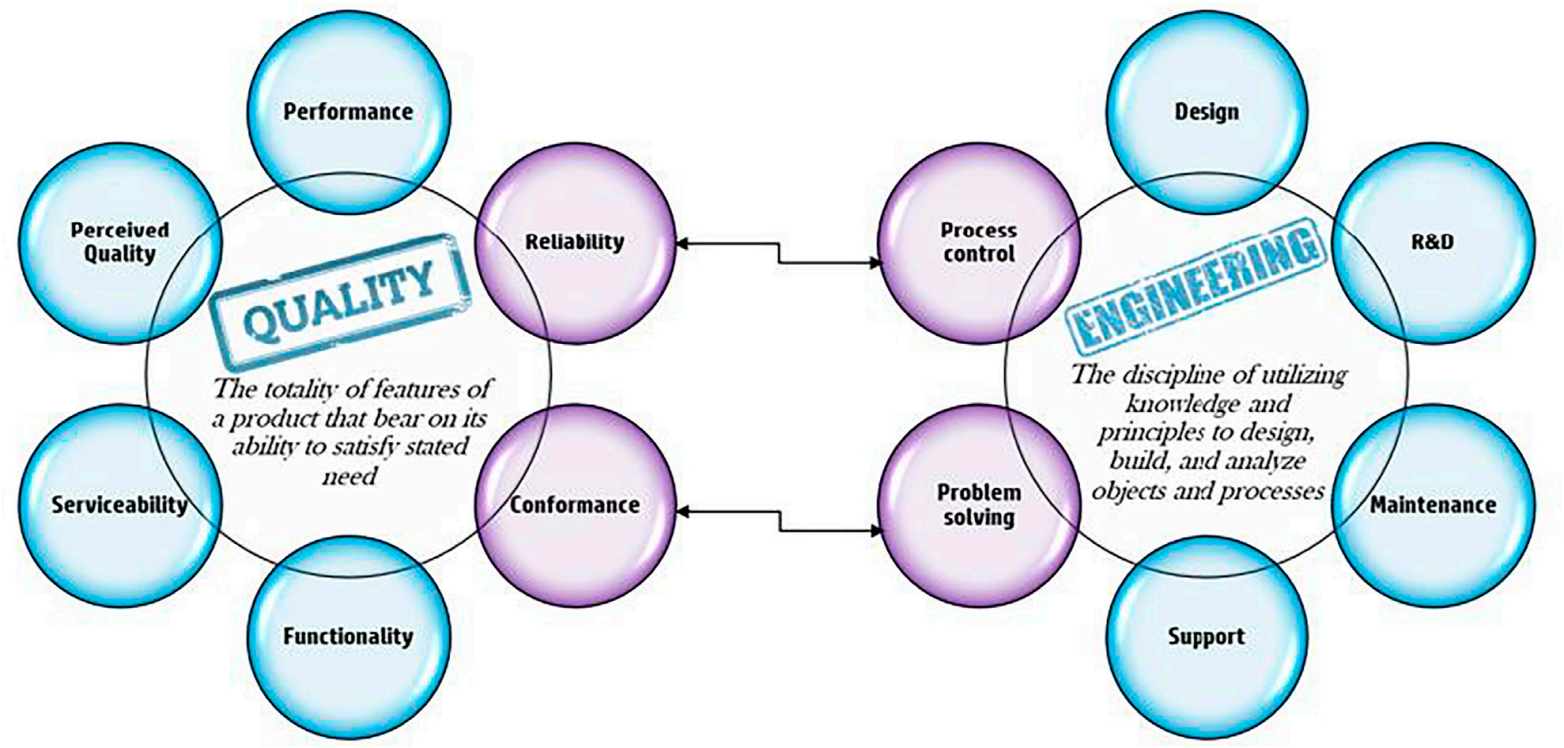
Quality and engineering strengths combined.

The goal of any business is profit and growth as the complexity of the business grows the holistic approach of looking on the wide picture and targeting a “global optimum” is getting more crucial. The concept of global optimum looking at the process as a system and working toward the optimal option for the entire system to reach better outputs is discussed as a part of the theory of constraints “*we are always talking about the organization as a whole, not local optimums*” (Goldrat 1988). The holistic view of the management process calls for an integrative approach as described in the work of Stanislav Karapetrovic (Karapetrovic 2002). From both these references, we gather that the need for a “seamless” process approach overlooking the “big picture” is essential in any organization’s path to success. This “seamless” process approach is where quality acts as the skeleton and it needs to include global functions throughout the supply chain (Das 2011). Deployment of strategic quality management method is also compared in the literature to the strategic principles of Sun Tzu’s “Art of War” (Sui Pheng and hui Hong 2005), as well as in a study by Raikov (2019), suggesting that strategic risk temperature (SRT) is a valid method for reducing risks in manufacturing organizations. From our own experience, two key engineering skills are process control and problem solving; adopting these skills to the world of quality enables the transformation of the classic quality indicators into a powerful strategic tool driving organization growth. Figure 2 illustrates the connecting areas between the engineering and quality disciplines. Despite the research mentioned above, process approach to quality management is hardly mentioned in the literature. In the industry, quality management is being done mostly through indicator control and not process control or process management in the world industry (Ravoy 2018). As specified earlier, all processes exist within a specific range of variation and as there is no such thing as a “perfect process,” we expect to have materials, parts, and products that will deviate from this range, that is, nonconformities (NCs). The first requirement for NC is in the ISO standard states—“*The organization shall ensure that product which does not conform to product requirements is identified and controlled to prevent its unintended use or delivery*” (BSI 2011). The two keywords here are “identification” and “control.” NC identification is an extension of the requirement for identifying all materials, parts, or products by suitable means throughout product realization. The standard, however, does not prescribe any particular methods of identifying nonconforming products. Indeed, it can take many forms, all of which have their place, for example, tags, labels, tapes or ribbons, codded markings, electronic identifications, and more. The control of the NC is the next requirement and that indicates the need for a process. In our opinion, it is possible to define NC management within an organization as a process with defined steps to be controlled and documented procedures. The first step in understanding a process is defining its goals, measures, and targets; these are usually strategic aspects, which have been defined “top down” by the organization management team and then broken down to each individual unit/function (Becker et al., 2014). Since the NC process is based on a negative output of the operational process, a part/material/product that does not conform to its requirement, the assumption is that the overall objective should be to reduce the output and its impact on the organization, and the most basic measure would be the number of items defined as NC.

**FIGURE 3:**
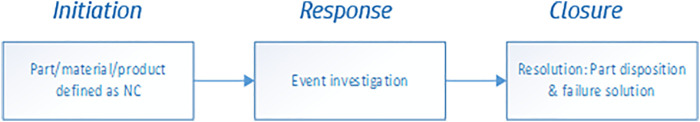
Basic NC process.

**FIGURE 4:**
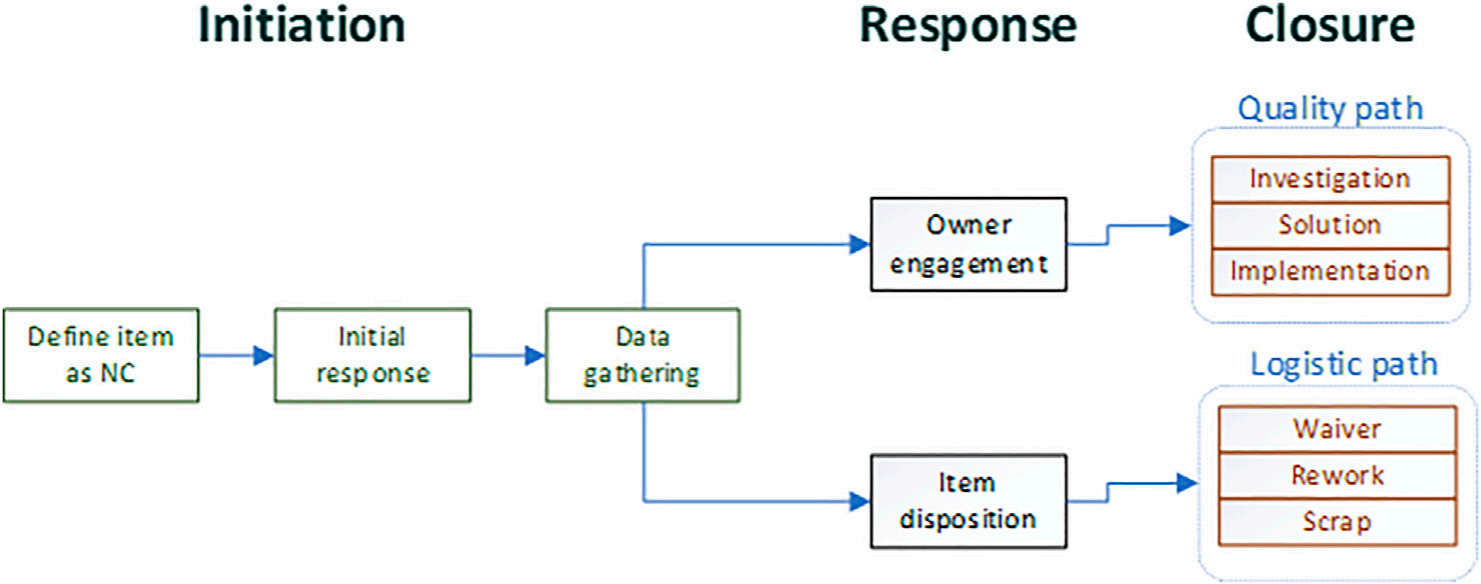
NC process phases.

As we defined previously, any process basically consists of three main parts—input, processing, and outputs. Applying this logic to the NC, our input will be the indication that if an item is nonconforming, the processing step will be the decisions and actions done with that item and the output would be a solution for the specific part and the reason for the nonconformity. Figure 3 gives a generic view of an NC process. In the processing part, there is a need to answer two questions: What to do with the rejected item ? How/Who will solve the problem? The answers to these questions expand the width of our process. The first question is pure logistic on what to do with the nonconforming part—we can repair it, scrap it, waiver the nonconformity, etc. The second question connects us to another key skill—problem solving. Solving the issue, that is, preventing the repetition of the event requires defining the gap, investigating the root cause, and developing a corrective action (Saad, Al-ashaab, and Shehab 2013). We claim that in order to better understand the process, we need to further break it down. The three steps described in Figure 4 can be defined as three phases—initiation, response, and closure. In the initiation phase, the event is being generated, that is, someone raises a “flag” that we have a nonconforming material/part, this phase should also include data gathering and initial response to the event, for example, stopping the line and putting material on-hold. In the second process phase, the NC is routed to a designated operational function, a function that will manage the flow in terms of the specific item and the overall event closure. This function can be an engineer correcting a design flaw or a procurement specialist updating supplier procedures. The main point in phase two is the relevant function acknowledging the event and taking ownership over it.

The third phase of the process is closing the item, that is, completing the logistic path for the item (disposition) and conducting the investigation leading to solution development. This last part can evolve into a full-scale problem-solving cycle and even an engineering design project. Figure 4 illustrates the NC process phases. An important term in this process is the generic material review board (MRB). The MRB is an optional tool in the process to deal with complex issues, usually the ones that involve several operational interfaces (Evans 2002). The definitions of the process flow refer only to the steps—what needs to be done, and not to the ownership aspects of it such as who should lead the effort? Who is responsible for the event origin? Or, who should pay for the repair/damage?

### Prioritization Methods and Their Relevance

Making managerial decisions is for directing the organization onto a planned strategy, and it usually involves balancing desired activities aimed at production, development, and growth to the constraint of available resources and funds. From this aspect, we can understand the importance of prioritizing the activities at hand and starting with the activities that will have the largest impact on our organization’s progress. The same applies when we have to choose a problem to fix an NC or to investigate. We believe that giving management a data-driven tool for making business decisions is in fact the realization of Deming’s vision that he outlined in his article published in 1986 that good managerial decisions need to be based on objective facts and numbers (Deming 1986).

As the process complexity increases, so does the scope of data associated with its nonconformities and again, we need to adjust our work based on Quality 4.0 concepts. The data can increase in several different vectors such as volume or variety; based on our experience in the world of nonconformities, the scope of large data variety is significant. This variety may manifest in the type of the NC or its impact size. The impact size of an NC can vary significantly as one can cause a major production stop, for example, and the other might just skew our production line within the regular production range. In order to prioritize correctly and drive real impact on the operation, we need to define a method of calculating the weights moving from “gut feelings” to data-driven process. In this aspect, prioritizing NC events is basically taking a list of events that can be referred to as transforming the qualitative data into quantitative data. Qualitative data, in its nature, are less objective than quantitative data, and in the transformation, we induce a certain level of uncertainty. In order to overcome and reduce this risk of mistakes, the key guideline is increasing objectivity. Increasing objectivity can be done by a broad scope of people, breaking processes into small details, bringing in “fresh eyes” to join the transformation. This kind of transformation is used in risk management when trying to estimate the risk size and prioritize it, a common method for this transformation is Failure Mode and Effect Analysis (FEMA). The basic principle in FMEA is defining three attributes that can support risk priorities and surveying these attributes to create a map of the assessment on a process or product (Subriadi, 2020). A similar technique can be adopted for other events of data transformation with the relevant attributes defined, such as waste reduction priorities (de Suza et al., 2014).

In the next section, we will review the research aspects of the topic, that is, the model NC prioritizing or the NC severity model. In order to focus on the research, we defined a very specific scope—only prioritizing NCs and not dealing with routing the parts, setting ownership, or solving the issue.

## Research Methods

The method in the research will be a case study and experts’ survey—developing, using, and measuring the model in one organization. The case study will take place in a large company from the printing business industry. In order to get a wider perspective on the issue of nonconformities management, prioritizing the research will also include a survey with various quality leaders in different organizations.

The main steps in building the case study were as follows:

1) Define the elements of the model calculations.
2) Plan a pilot with clear success criteria.
3) Plan of model implementation (pending successful pilot conclusion).

An important aspect of the model buildup is alignment between the stakeholders, as NC management affects different units in the organization (manufacturing, procurement, engineering, logistics, and more). These stakeholders were part of defining the model as the overall objective is for the entire organization.

### Defining the Model

Nonconformity is one of the eight types of waste (Linker and Meier, 2006) as it is a product/part/material/operation defect slowing the operations and requiring extra/unplanned resources. Waste such as defects bears a direct impact on the company’s financial bottom line and is referred to as the cost of quality, cost of poor quality to be more precise (Teli et al., 2013). The impact of each NC, as previously explained, is not constant, and this leads to the need for ranking and prioritizing.

**FIGURE 5:**
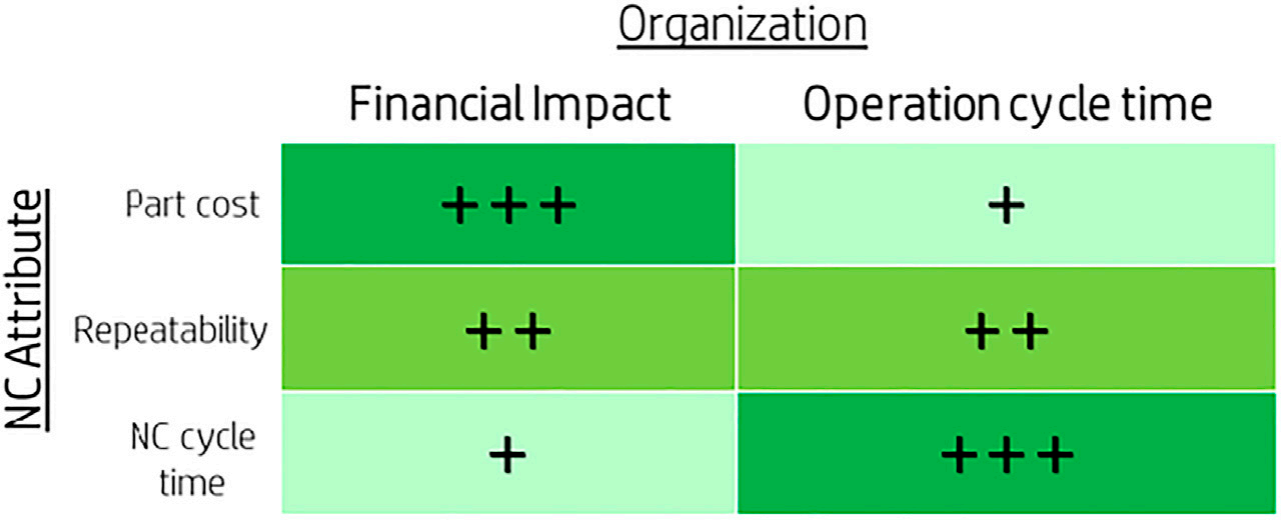
NC attributes and their relative impact (+minor; ++medium; +++high) on the organization.

**FIGURE 6:**
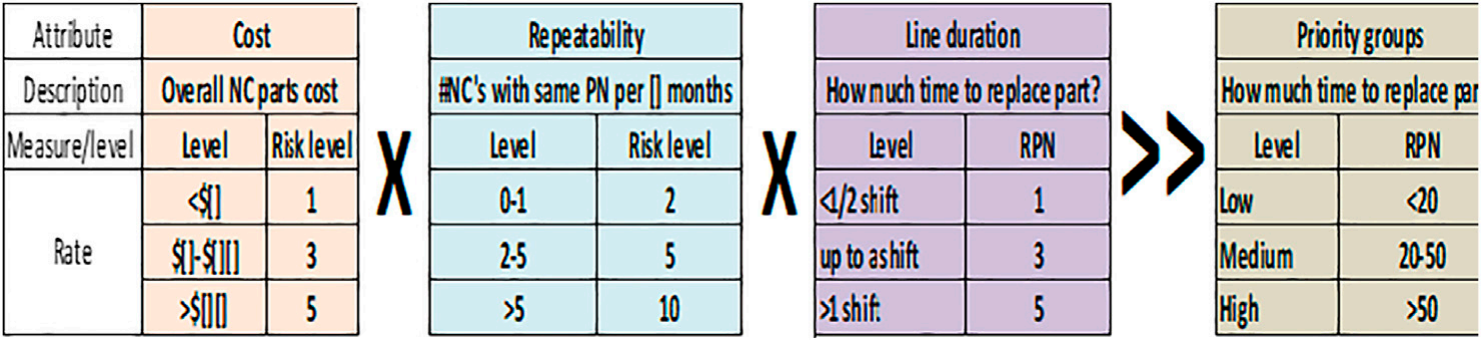
NC priority calculation (RPN = risk priority number).

Relevant vectors impacting the organization’s “bottom line” (financial impact and operation cycle time) were chosen as the attributes for the prioritization model. Figure 5 illustrates the connection between the chosen attributes and the organization’s outcomes. Taking these attributes, we defined a calculation for a “priority number” similar to the RPN (risk priority number) used in the FMEA risk assessment method, thus creating a “ruler” of NC ranking. Again, based on the parallelism to the FMEA risk management method, once we establish the priority number, we sort the NCs and group them into three main levels—high, medium, and low priority. The model drives the organization into a continuous improvement cycle—high-priority NCs are raised and handled first, and the next batch of issues is raised next. An important aspect for the model success, as any action taken within a business organization, is the balance between the improvement planned and the resources needed. In this case, the model needs to highlight a group of events which the organization can handle, and the aim was set on having ∼20% high severity events. Figure 6 describes the calculations defined for the NC severity model in order to set a priority number for each attribute and an overall number for the event. The severity number is called RPN (risk priority number) as done in the FMEA methodology. The logic behind the numbers placed in the initial model settings was to have a clear definition between the three levels of severity in a type of logarithmic scale (Paciarotti et al., 2014). The joint development work of the stakeholders defined that a repeating event bears more impact than the other attributes and that is why it was given higher weight in the model (see also in Figure 6).

### Model Pilot and Implementation

**FIGURE 7:**
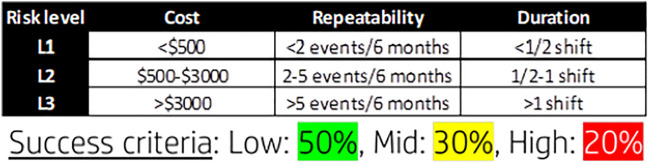
Pilot initial thresholds and success criteria.

A part of any development process is testing and proving the research hypothesis (Saunders et al., 2012), in this case proving by using the NC severity model will direct us to the high-priority group that the organization resources can handle. The test pilot was designed to direct the organization into 20% of high-priority NCs based on the initial knowledge of the team. The scope of the pilot was 1 month. Figure 7 shows the initial thresholds of the pilot and its success criteria. The model pilot was planned to run in three different production lines.

Following the model pilot and pending successful results, the model will be implemented into the organization’s NC routines. The model is supposed to be added to the company’s managerial routines.

The implementation concept consists of the following main steps:

1) Manual use of the model in all production lines (excel forms) for three quarters
2) Control of model impact via:
  a NC standard managerial reviews.
  b Adding top NCs list based on the model to the NC dashboard.
3) Drive structured problem solving via A3 methodology (Saad, Al-ashaab, and Shehab 2013) based on the top NCs list.
4) Promoting Quality 4.0 approach by developing automatic calculations for the model within the quality reports system

### Quality Experts Survey Plan

As stated previously, nonconformities management is a generic part of almost any operation; the objective of the survey was to get different perspectives on it from different organizations. It is to be noted that the survey itself is not part of the research case study and statistics.

The scope of the survey covered quality leaders from seven different companies spread over five different countries. The survey was done using a questionnaire with the following sections:

1) “Contact ID”—basic details of the contact person including job title and relevant quality certifications.
2) “Company”—understanding the nature of the company (operation type, classification, quality location within the company, etc.).
3) “Nonconformities management”—specific questions on the NC process (definitions, prioritization, etc.).
4) Free text section for additional comments.

**FIGURE 8:**
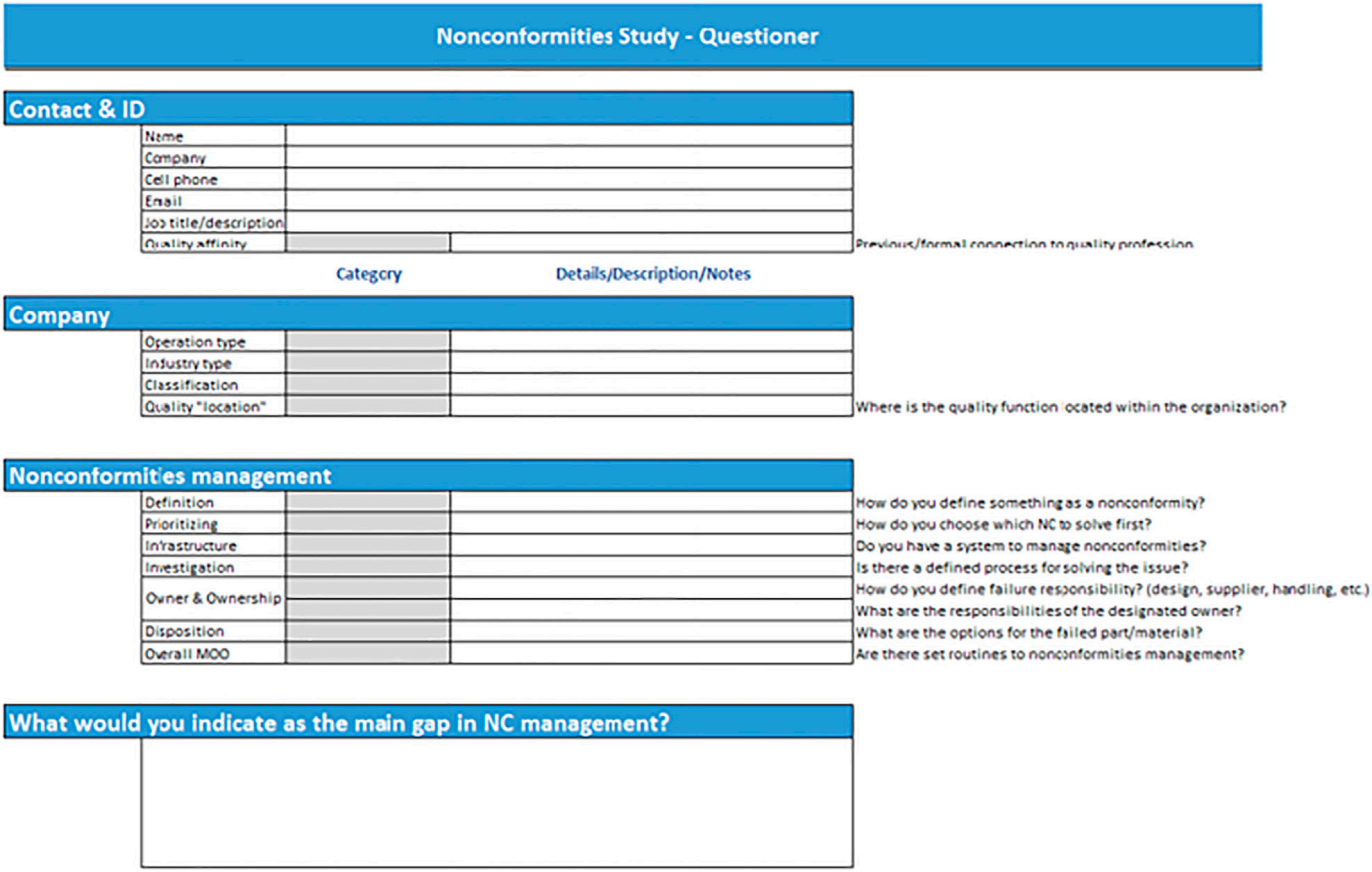
Experts’ survey questionnaire.

Figure 8 presents the template of the questionnaire that was used in the quality experts survey.

## Results and Discussion

### Pilot Outcomes

**FIGURE 9:**
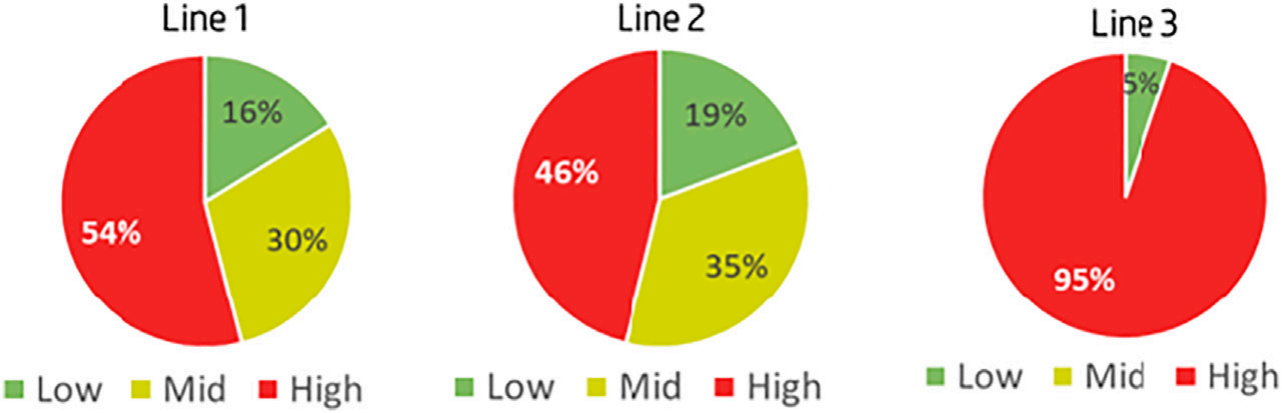
Pilot’s first iteration results.

The pilot was implemented with the settings defined in Figure 7 and the outcomes of NC distribution in the production lines did not meet the success criteria, directing to 20% high-priority NCS. Figure 9 shows the ranking results of the pilot’s first iteration, that is, the model settings were needed to be adjusted to align with the organization’s resources. Adjusting the model was also done as a joint work with the stakeholders putting more emphasis on the previous data from the manufacturing lines. Three types of changes were devised:

1) Each production line is behaving differently—need to set different thresholds for part cost and repeatability per production line.
2) Counting repeatability over a period of 6 months is too wide—measure repeatability over a timeframe of 3 months.
3) Optimize the matrix for defining the overall priority number per event (the consolidation of the priority numbers of cost, repeatability, and duration).

**FIGURE 10:**
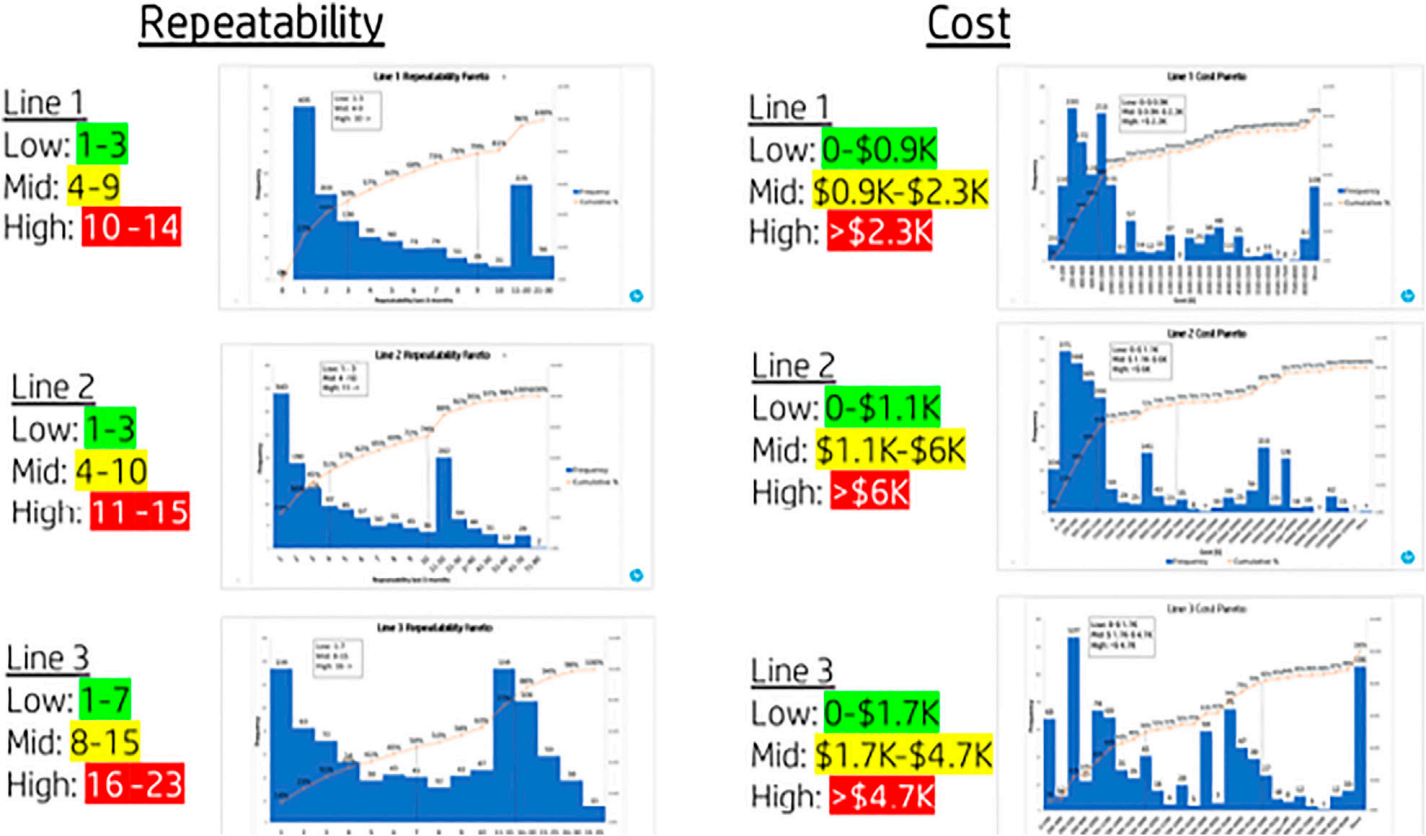
Model adjustment, setting specific thresholds per production line.

In order to set different thresholds for each production line, the previous population of NCs was reviewed in terms of cost and repeatability. Figure 10 summarizes the distribution analysis leading to the new model cost and repeatability values. The used method was charting a Pareto for each production line, and the three levels were set using the 50 and 80% points for each line.

**FIGURE 11:**
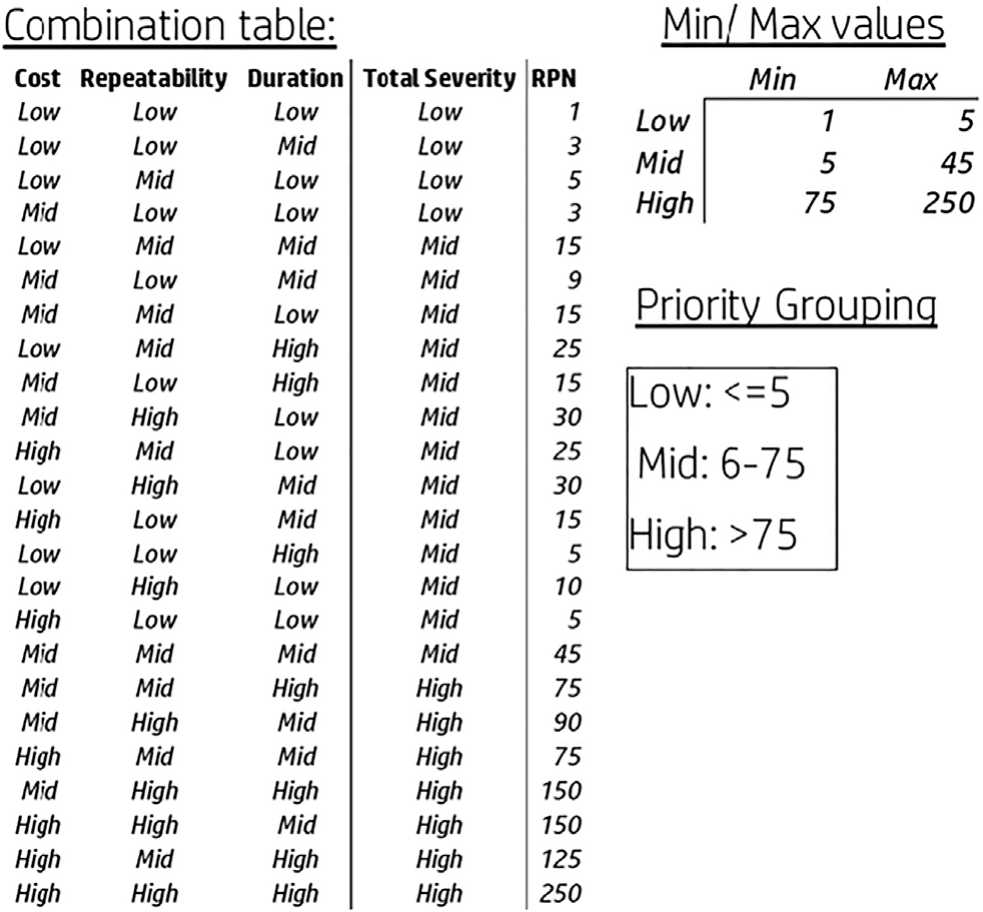
Model adjustment, priority grouping.

**FIGURE 12:**
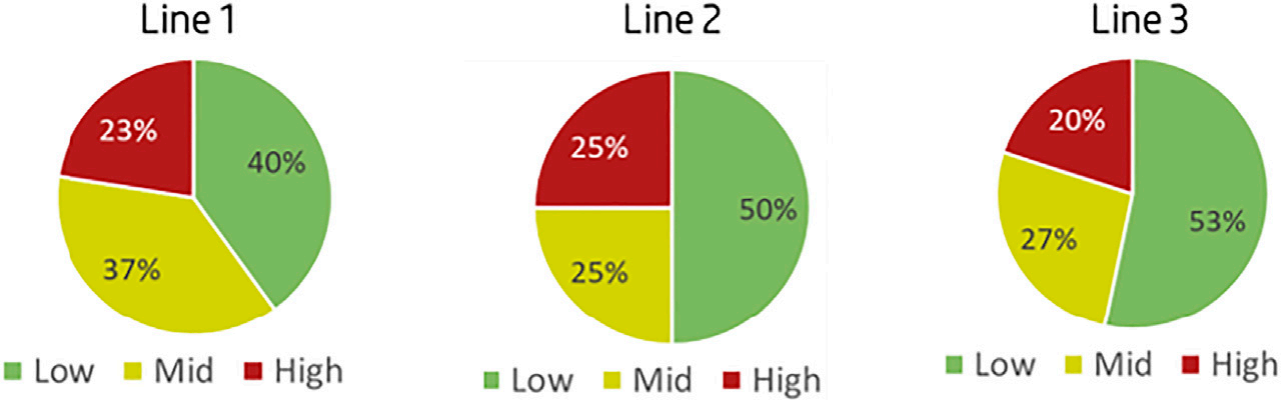
Pilot’s second iteration results.

As mentioned in addition to setting the specific threshold per production line and adjusting them the priority grouping, Low-Medium-High, was redefined based on the matrix in Figure 11. Following the model adjustments, the pilot was repeated, this time meeting the criteria of having ∼20% high-priority NCs as seen in Figure 12. The results of the pilot’s second iteration allowed for the initiation of the implementation plan into the production lines and the NC management routines as per the defined plan.

### Model Implementation and Results

Following the NC priority model’s successful pilot, the model was implemented in all of the manufacturing lines. The execution part took 3 months and consisted of the following elements:

1. The NC severity ranking was translated into a “top NC” list in the process management dashboard and forums.
2. On-going sanity check was initiated with all the stakeholders verifying continuous computability, in other words, making sure no top issues are being missed.
3. Part of the attributes are still manually reported and need to be automated in the next phase of process improvement.

The short list of “top NCs” enabled the quality team to promote an organization-wide move using a structured problem-solving method, and that in turn, resulted in a reduction of NC impact (Saad, Al-ashaab, and Shehab 2013). The main example was in the third quarter of 2020, where one manufacturing line had higher quantity of NC events, but since the top NCs were prioritized, the financial impact on that line was reduced. The model concept of utilizing operation attributes and calculating the priority based on proven risk management method gained confidence, and additional units are now exploring the possibility of adopting it to additional production lines, types of failures, and even engineering cycle time improvement programs. During the model implementation, there were also some new products introduced into the production line (NPIs), for the model was less effective in calculating the top events as the repeatability attribute was less available (different versions, development changes, etc.).

### Quality Experts Survey Summary

**FIGURE 13:**
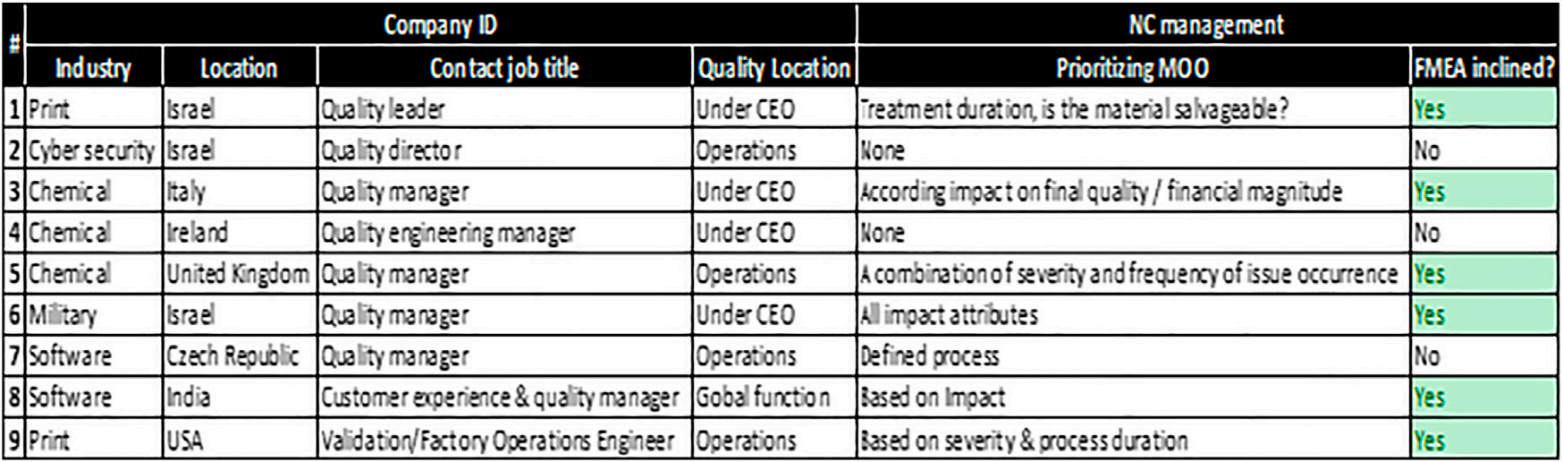
Survey results.

The quality expert survey incorporated feedback from quality leaders in nine different organizations spread over seven different countries. The aim of the survey was to get additional perspective on how NCs are being managed and more specifically prioritized. Figure 13 summarizes the main results from the survey. The survey clearly showed that quality managers tend to use at least part of the FMEA risk assessment concepts in prioritizing nonconformities as two-thirds of them indicated that they utilize attributes such as severity, cost impact, or duration impact. Some of the survey subjects commented on the fact that NCs are not prioritized in a structured process, leading to the misuse of resources and solutions having low impact on the business.

## Discussion

Utilizing the Quality 4.0 concept of grouping high-volume data by specific definitions and focused utilization, the model implementation drove a cultural change moving from reactive to proactive quality approach. The reactive approach defines all the NCs as equal, requiring the same level of attention, thus none of them can be thoroughly solved, while the proactive approach promoted by the NC severity model highlights the NCs with the higher impact and allows the organization to utilize its resources better. The transition is reactive to the proactive with the help of the proposed model that uses existing resources in the organization; therefore, this does not constitute a burden and only optimizes the activities of the organization.

The model was more effective on the “legacy” of product manufacturing line, where all the needed attributes are available and less effective for new products transferred from development into production. We learned that planning the model needs to be based on the previous data as done in the second pilot iteration when the Pareto analysis brought the model settings to its required criteria. In order to keep moving forward to continuous improvement, the model needs to be constantly updated, keeping the 20% high-priority threshold with a trend of reduction in the priority number itself.

As stated earlier, this model only deals with the initial part of the NC process—defining which issue to start with. It does not cover main elements such as who should solve the issue and how to control the overall process. These are the major topics for further research in order to create an optimized set of tools for the NC process.

## Data Availability

The original contributions presented in the study are included in the article/Supplementary Material, and further inquiries can be directed to the corresponding author.
